# The spatial profile of skin indentation shapes tactile perception across stimulus frequencies

**DOI:** 10.1038/s41598-022-17324-7

**Published:** 2022-08-01

**Authors:** Roman V. Grigorii, J. Edward Colgate, Roberta Klatzky

**Affiliations:** 1 Department of Mechanical Engineering, Northwestern University, Evanston, IL 60208 USA; 2 Department of Psychology, Carnegie Mellon University, Pittsburgh, PA 15213 USA

**Keywords:** Neuroscience, Engineering

## Abstract

Multiple human sensory systems exhibit sensitivity to spatial and temporal variations of physical stimuli. Vision has evolved to offer high spatial acuity with limited temporal sensitivity, while audition has developed complementary characteristics. Neural coding in touch has been believed to transition from a spatial to a temporal domain in relation to surface scale, such that coarse features (e.g., a braille cell or corduroy texture) are coded as spatially distributed signals, while fine textures (e.g., fine-grit sandpaper) are encoded by temporal variation. However, the interplay between the two domains is not well understood. We studied tactile encoding with a custom-designed pin array apparatus capable of deforming the fingerpad at 5 to 80 Hz in each of 14 individual locations spaced 2.5 mm apart. Spatial variation of skin indentation was controlled by moving each of the pins at the same frequency and amplitude, but with phase delays distributed across the array. Results indicate that such stimuli enable rendering of shape features at actuation frequencies up to 20 Hz. Even at frequencies > 20 Hz, however, spatial variation of skin indentation continues to play a vital role. In particular, perceived roughness is affected by spatial variation within the fingerpad even at 80 Hz. We provide evidence that perceived roughness is encoded via a summary measure of skin displacement. Relative displacements in neighboring pins of less than 10 µm generate skin stretch, which regulates the roughness percept.

## Introduction

Almost a century ago, David Katz argued that the tactile perception of texture depends upon both a “spatial sense” for coarse textures and a “vibration sense” for fine textures^1,2^. This idea, widely known as the duplex theory, has stood the test of time. For example, putative neural codes based largely on the spatial distribution of SA-I and RA mechanoreceptor responses have been shown to predict the subjective roughness of coarse textures^3,4^, while precise spike timing associated with PC afferents has been shown to correlate with perceptual aspects (e.g., roughness and similarity) of fine textures^5,6^. A number of recent studies, however, have begun to reveal limitations to the duplex theory. For instance, it has been shown that faithful reproduction of skin vibrations is insufficient to reproduce the feel of a natural texture, or even the roughness of the texture; moreover, this was found to be true for a variety of both fine and coarse textures^7,8^. Indeed, when comparing a real texture with a stimulus that produces the same vibrations on the skin, subjects needed to scale the latter by a factor of 3-5 in order to match perceived intensity^7^. Additionally, it has been shown that the spectra of skin vibrations produced when exploring fine texture are highly sensitive to conditions of touch, such as scan speed and pressure, yet the percepts themselves are quite stable^6,9^. These findings suggest that temporal encoding of bulk skin vibration may not be an adequate explanation of fine texture perception. Some form of spatial encoding may take place on finer texture scales than previously thought, warranting a deeper investigation into the fundamental aspects of texture perception.

One of the difficulties of studying texture is achieving precise control of the mechanical stimulus at the surface of the skin. A common approach is the careful design or selection of textured surfaces in order to exert some control over the pattern of fingerpad displacement or vibration during sliding contact^4,5,10^. This approach, however, must contend with the highly complex interfacial mechanics of skin-surface interaction, leaving the essential features of the peripheral excitation somewhat opaque^11,12^. In order to avoid modeling or measuring the complex mechanics of sliding skin contact and to achieve more precise control of the peripheral excitation, an alternative approach is to actuate the skin with a transducer-driven system and then relate the known actuation parameters to perception^13,14^. This is the approach that we have taken by developing a custom pin array that provides a degree of control over both spatial and temporal patterns. The array includes 14 individually-actuated pins that provide normal indentation of the skin at frequencies from 5 to 80 Hz. The pins are arranged in a hexagonal pattern with 2.5 mm centers. Using this array, we aim to: (1) measure the detection thresholds associated with spatial variation of skin indentation at a number of excitation frequencies and (2) relate the spatio-temporal parameters of skin excitation to higher-level tactile experience. In doing so, we hope to shed new light on the manner in which Katz’s “spatial sense” and “vibration sense” may work cooperatively to inform the tactile sensation that is texture.

## Results

**Figure 1:**
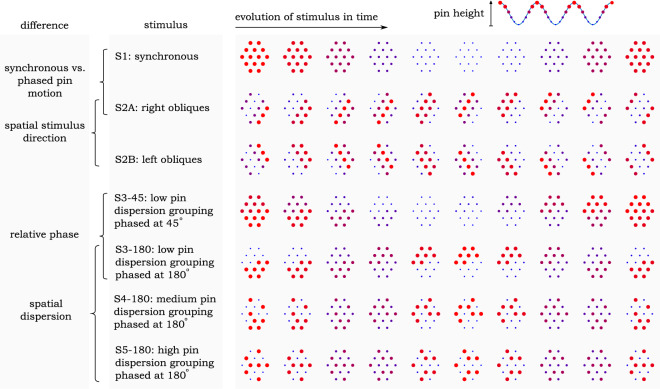
An illustration of single cycles of stimulus evolution as a function of time across several stimulus categories used in the experiments. S1, S2A, and S2B are stimuli used in Experiment 1. S3-x, S4-x, and S5-x stimuli, where x represents relative phase of two pin groupings, are used in Experiments 2 and 3. Note that S1 and S3-x, S4-x, and S5-x are the same stimulus when x = 0. Further note that the spatial wavelength of S2A and S2B stimuli was set to 8 mm.

Psychophysical studies were conducted with several spatial patterns of pin motion and thus skin displacement. These patterns were composed by driving all 14 pins at a fixed amplitude and at a single frequency in the 5–80 Hz range, but varying the relative phase across the array (Fig. 1). Using these stimuli, we conducted a series of experiments that tested for (1) discrimination of all pins moving in synchrony (S1) versus a shape-based pattern (S2A) in which a wave propagates under the finger, as well as discrimination of two shape-based patterns (S2A and S2B) that differ only in direction of wave movement; (2) the conditions under which the asynchrony in motion of neighboring pins is perceptible (discriminating S1 vs S3-x, and S1 vs S5-x); and (3) the effect of frequency, phase delay, and spatial distribution in relative motion of neighboring pins on perceived roughness—a textural feature (comparing S3-x, S4-x, and S5-x). In the first two experiments, participants were tested under the 3AFC psychophysical protocol, where they were asked to select the odd one of three presented stimuli. In the third experiment they performed free magnitude estimates of perceived stimulus roughness.

**Figure 2:**
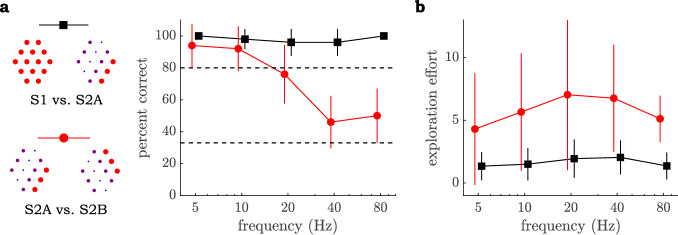
Results from Experiment 1. (**a**) Participants’ identification performance in the 3AFC task contrasting S1 vs S2A stimuli (black square) and S2A vs S2B (red circle). Chance identification and the probability associated with *p* = 0.05 statistical significance (derived under a binomial distribution) are shown as dashed lines. (**b**) Exploration effort during the identification task, represented by the average number of switches between stimuli beyond the minimum of two. Lines represent standard error. Exploration effort between two tasks is significantly different at all frequencies (*p* < 0.001).

Results from the first experiment are presented in Fig. 2a as a function of pin frequency. It is evident that the S1 and S2A stimuli are perceptually distinct at all frequencies tested here, suggesting that deviation from uniform skin displacement is a readily perceptible cue. S2A and S2B, in contrast, are distinguished at the criterion of statistical significance only at frequencies up to about 20 Hz, suggesting that the perception of shape-related stimulus features is limited to those lower frequencies. We note that above-chance discrimination between S2A and S2B exists at 40 Hz and 80 Hz; however, this may have occurred due to slight differences in the distribution of skin pressure across the pin array that provided uneven forces to input pin displacement. It is unlikely that shape information itself was communicated at these frequencies. Figure 2b displays the exploration effort required to identify the odd stimulus out of the three. Two switches between stimuli would be the minimum needed during a single trial; accordingly, effort was measured by the average number of additional switches beyond the initial two. An ANOVA with factors comparison and frequency found that significantly more switching occurred when comparing S2A vs S2B than S1vs S2A, (F(1,9) = 20.30, *p* < .001), and this effect was seen across frequencies.

These findings illustrate the difficulty of the S2A vs S2B comparison, which relies entirely on shape information (i.e., the orientation of the oblique line forming the wave). If we conclude that global, shape-based information is available only at the lower frequencies, then the ease of differentiation between S1 and S2A at higher frequencies must be due to another mechanism. We propose that at these frequencies, participants utilize summary-based information related to texture. An example of a summary-based measure is the sum over the array of the relative displacements of neighboring pins (unsigned and time-averaged), which would distinguish S1 from S2, but not S2A from S2B. To investigate this more closely, we performed additional experiments intended to isolate summary-based mechanisms by focusing on frequencies > 20 Hz.

**Figure 3:**
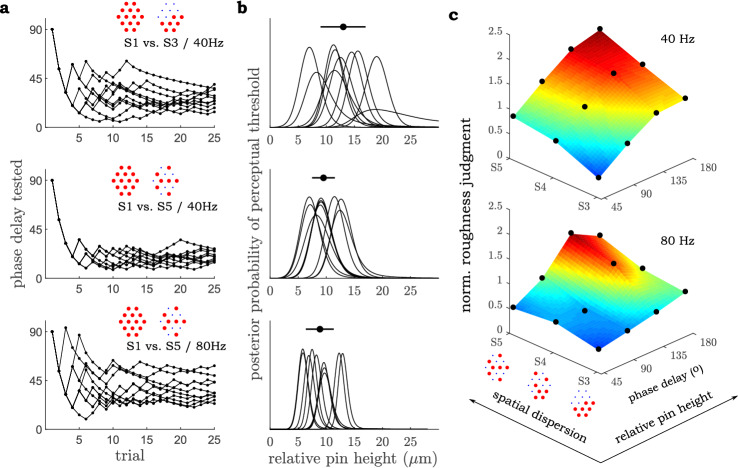
Results from Experiments 2 and 3. (**a**) Adaptive threshold measurements of pin phase for all subjects. (**b**) Posterior distributions of possible threshold values of relative displacement. Solid circles represent the mean of the peak location of each posterior distribution, and the horizontal line represents the associated standard deviation. (**c**) The effect of phasing of two pin groups and the dispersion of pins within these groups on perceived roughness.

Results from the second experiment are shown in Fig. 3. Because pin motion is sinusoidal, perceptual responses are considered in relation to parameters of the sine function: amplitude (A), frequency (f), and phase (*x*), A·sin(2*π*ft + *x*). Results indicate that at 40 Hz actuation, a 32° phase delay between two groups of pins in the S3 configuration (Fig. 1) can be distinguished from uniform motion, i.e. the S1 stimulus. This phase delay corresponds to a 13 µm peak relative skin displacement between pins in out-of-phase motion. Importantly, pins in the higher dispersion S5 configuration can be distinguished from S1 with a phase delay of 19° at 40 Hz and a delay of 37° at 80 Hz, which correspond to 9.5 µm and 8.9 µm relative displacement respectively (Fig. 3b). This threshold value in S5 is notably smaller than in S3 (*p* < 0.005), indicating that the spatial distribution of pin grouping has an effect on perceptibility, such that pin grouping with higher spatial dispersion leads to smaller overall threshold displacement associated with detecting deviation from uniform skin indentation.

The results of the third experiment, which tested the influence of the spatial dispersion of pin grouping and the phase delay of pin motion between these groups on perceived roughness, are presented in Fig. 3c. Clearly, the pattern by which pins are excited across the fingerpad has perceptual consequences, even when the frequency and amplitude of their motion are kept the same. Both the relative displacement of distributed points of excitation and the spatial distribution of these points are found to contribute to scaling perceived roughness. Given prior evidence that global shape information is not communicated at the tested frequencies, these results indicate that an aggregate or summary characteristic dependent on the spatial distribution of skin displacement has a pronounced effect on one of the fundamental perceptual qualities of texture^15^.

We next consider which of the three mechanically sensitive receptors (SA-I, RA, and Pacinian (PC)) is likely responsible for our perceptual findings and what mechanism underlines their function of detecting relative pin motion. Prior research suggests that neither the SA-I or PC mechanoreceptive population is appropriate. A factor pointing away from PCs is that the observed relative displacement threshold, which is essentially flat from 40 to 80 Hz, is not characteristic of the PC sensitivity curve, which tends to decrease rapidly over this frequency range^16^. Another factor is that the threshold of ≈ 10 µm found at 80 Hz is at least three times larger than that observed in PC populations^17^. The relatively high spatial acuity of the SA-I afferent may make it a plausible candidate to explain the sensitivity to spatial dispersion seen here, but threshold values are reported to be significantly higher (≈ 100 µm^17,18^) than those found in exp. 2 or those used to generate stimuli in exp. 3, namely 14 µm and 28 µm. The RA mechanoreceptor, in contrast to PC and SA-I populations, is a strong candidate to account for the observed roughness differentiation in response to temporal and spatial variation of skin indentation. The RA population is sensitive to relative local displacements of skin tissue^3,4,13^; it has high spatial innervation density (1.4 units/mm^2^) with correspondingly small receptive fields; and it has an estimated threshold response level consistent with the threshold found here^19–21^. In addition, temporal responsiveness to transient stimuli is a feature of this population^22^. To further investigate how the present display would activate the RA population based on the mechanics of skin indentation it induced, we evaluated a set of models that predicted the observed variations in roughness in response to frequency, phase, and spatial dispersion.

### Modeling

Four candidate summary-based predictors were selected based on the display mechanics, and each was found to be correlated with the mean values of reported roughness across subjects. (Ratings of roughness were also consistent across individuals; the median pairwise inter-correlation coefficient of roughness ratings is r = 0.78). The predictors were: skin normal deformation, skin stretch, and the rate of change of each. In all cases, these values were determined for each pair of neighboring pins, then absolute-valued and averaged across a cycle, and finally summed across all pairs of pins. Note that we assume neighboring pins moving in phase with one another produce no relative skin displacement or stretch between their points of contact with the skin. Further note that, due to ill-defined boundary conditions, we do not consider skin displacement outside of the pin array in our models. We expect that the contribution to summary displacement and stretch from the perimeter of the pin array will be constant across all stimuli, and thus will not affect our model fits or the subsequent conclusions drawn from them.

**Figure 4:**
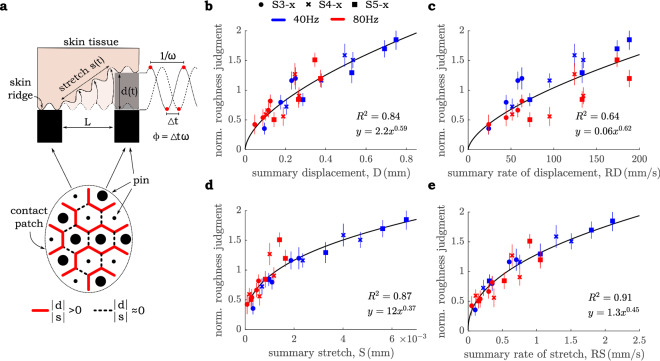
The mechanics model and the associated summary predictors. (**a**) Vertical pin positions within the fingerpad in the S5-x, x = 180° pin configuration. Sizes of solid circles within the contact patch correspond to the vertical pin position, as demonstrated by a side view of skin indentation profile (not drawn to scale). Lines separating each pair of neighboring pins represent relative tissue displacement: near zero for pins of the same phase, and above zero for pins of differing phase. (**b**–**e**) Average roughness judgments (error bars represent standard error of the mean) plotted against the time-average of summarized absolute predictors, as follows: (**b**) Displacement from Eq. (1); (**c**) Rate of displacement from Eq. (2); (**d**) Stretch from Eq. (4); (**e**) Rate of stretch from Eq. (5).

*Displacement (D)* The first roughness predictor is based on the relative skin displacement between neighboring points of skin to pin contact. For each pair of neighboring pins, we compute the absolute relative displacement, d(t) (Fig. 4b), integrate over time, and sum over all 29 neighboring pin pairs:

1 $$  {\text{D}} = \frac{1}{{\text{T}}}\mathop \sum \limits_{{{\text{k}} = 1}}^{{29}} \mathop \int \limits_{0}^{{\text{T}}} \left| {{\text{d}}_{{\text{k}}} \left( {\text{t}} \right)} \right|{\text{dt}} $$

where T is the oscillatory period.

*Displacement Rate (RD)* The second predictor is similar to the first, but based on the relative rate of displacement of neighboring pins:

2 $$  {\text{RD}} = \frac{1}{{\text{T}}}\mathop \sum \limits_{{{\text{k}} = 1}}^{{29}} \mathop \int \limits_{0}^{{\text{T}}} | {\frac{d}{{dt}}{\text{d}}_{{\text{k}}} \left( {\text{t}} \right)} |{\text{dt}}  $$

*Stretch (S)* Relative pin displacement and its rate may not be the natural input to the transfer function of a given mechanore- ceptor, since at the local level it is tissue stretch surrounding the RA receptor that produces a response^23^. We therefore approximate the local skin stretch generated by out of phase pin motion, s(t), as the difference in distance between the tips of any two neighboring pins when positioned the same height, which is set by the array pitch of L = 2.5 mm, and the distance between the pin tips when one leads the other by relative displacement d(t), namely $\sqrt {{\text{L}}^{2}  + {\text{d}}\left( {\text{t}} \right)^{2} }$ Fig. 4a):

3 $$  {\text{s}}\left( {\text{t}} \right){\text{~}} = \sqrt {{\text{L}}^{2}  + {\text{d}}\left( {\text{t}} \right)^{2} }  - {\text{L}}  $$

The summary of the absolute stretch can then be approximated by:

4 $$  {\text{S}} = \frac{1}{{\text{T}}}\mathop \sum \limits_{{{\text{k}} = 1}}^{{29}} \mathop \int \limits_{0}^{{\text{T}}} \left| {{\text{s}}_{{\text{k}}} \left( {\text{t}} \right)} \right|{\text{dt}}  $$

*Stretch Rate (RS)* Similarly, summary of the absolute rate of local stretch can be found by:

5 $$  {\text{RS}} = \frac{1}{{\text{T}}}\mathop \sum \limits_{{{\text{k}} = 1}}^{{29}} \mathop \int \limits_{0}^{{\text{T}}} | {\frac{d}{{dt}}{\text{s}}_{{\text{k}}} \left( {\text{t}} \right)} |{\text{dt}}  $$

Table 1 reports R^2^ values for linear and power functions fit to the roughness ratings under each of the four models tested. Perceived roughness was found to scale closely with the magnitude of summary skin displacement, stretch, and their respective rates of change (Fig. 4b, c, d, and e), confirming our hypothesis that distributed skin displacement has a pronounced effect on perceptual experience. However, the best overall fit was provided by a power function relating participants’ assessment of stimulus roughness to the summary rate of skin stretch (Fig. 4e), accounting for 91% of the variance when fit simultaneously to both frequency levels used in the experiment. Unlike competitors, separate fits at 40 Hz and 80 Hz with this model yielded similar parameters. These results are in line with previous research that points to rate of skin deformation to be the best predictor of RA afferent responses^18,24^. Notably, the model based on rate of skin stretch incorporates not only the local effects of pin displacement, but also the interactions between nearby points of displacement summarized across the entire contact patch.

**Table 1:** R^2^ values for linear and power functions fit to each of the predictors in (Fig. 4).

Fit type/data used	D	RD	S	RS
Linear/40 Hz	0.84†	0.84†	0.89‡	0.91‡
Linear/80 Hz	0.73*	0.73*	0.79*	0.79*
Linear/40 Hz + 80 Hz	0.82‡	0.64†	0.78‡	0.87‡
Power/40 Hz	0.86†	0.86†	0.97‡	0.97‡
Power/80 Hz	0.71*	0.71*	0.77*	0.77*
Power/40 Hz + 80 Hz	0.84‡	0.64†	0.87‡	0.91‡

Separate functions are reported for each frequency as well as two frequencies combined. Significance level is denoted as ‡: *p* < 0.0001, †: *p* < 0.001, ∗ : *p* < 0.01.

## Discussion

The present experiments inform our understanding of texture perception in multiple ways. A fundamental finding is that frequency of vibration on the skin appears to play an important role in determining what aspects of texture are conveyed. Local skin indentation in the 5–20 Hz range generates cues that allowed participants to make discrimination based on the overall spatial pattern of stimulation, i.e. shape. In contrast, in the 40–80 Hz range, shape cannot be discerned, but skin stretch and the rate of its variation continue to inform the percept of roughness. A dual perceptual mechanism thus appears to be at play—one operating on the global qualities of the stimulus, which appears to be band-limited to 20 Hz, and the other on the summary information derived from fingerpad excitation. Our data indicate that perception of roughness via this latter mechanism is mediated by relative displacements of skin tissue within the fingerpad down to 9 µm ± 2.3 µm. This finding is consistent with measurements of perceptual thresholds as well as RA response thresholds at 10 µm, leading us to believe that this mechanoreceptor is responsible for encoding spatially distributed and highly transient excitation within the fingerpad. Importantly, roughness is well accounted for by only those models of skin/surface interaction that incorporate both the magnitude and spatial dispersion of indentation, the latter serving the role of increasing instances of localized stretch through differential deformation. Finally, assuming stretch is indeed the primary factor in determining RA response, 9 µm deformation applied to Eq. (3) would suggest that skin stretch on a nanometer scale is sufficient to trigger perceptible cues, thus indicating the potential significance of spatially distributed skin stretch in the perception of very fine surface features.

**Figure 5:**
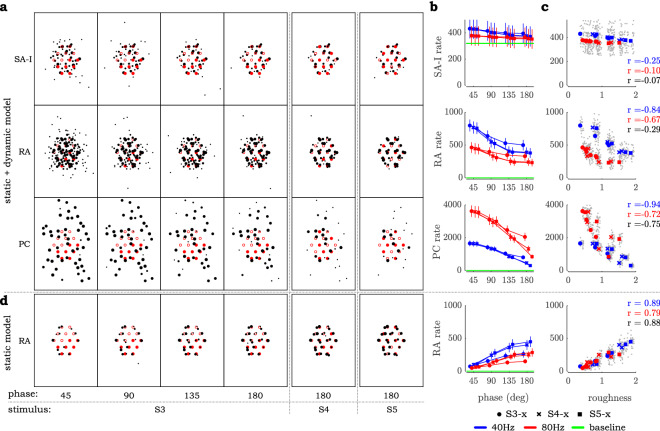
Results of TouchSim (TS) simulation with 1.2 mm base skin indentation and the same vibration amplitudes used in Experiment 3. (**a**) Spatial maps of afferent activity as measured by mean firing rate for each afferent (scaled black dots) relative to pin placement (red open and closed circles denote the two groups of pins) across a 10-s run. (**b**) Mean afferent firing rate, summed across the afferent population, plotted alongside base firing rates due to static pin indentation. (**c**) Firing rate as computed in (**b**) plotted against human roughness judgments from Experiment 3. Each grey point is the response of a single afferent over 20 10-s runs. Colored points are averaged across 20 runs, and r-values from correlations between rate and roughness across 20 simulations are coded by frequency (blue = 40 Hz, red = 80 Hz, black combined frequencies). (**d**) Spatial maps and mean RA firing rates generated by the TS simulation with only the static model component (dynamic wave propagation is disabled).

Although the models we tested focus on the mechanical interactions between pin display and the skin, questions about the neural consequences of these interactions naturally arise. For instance, it may be the case that mechanoreceptors are excited in direct relation to spatial variation of skin indentation^13,25^. To examine this at the neural level, we used the TouchSim (TS) software (as detailed in ‘Methods’) to predict SA-I, RA, and PC afferent spike patterns in response to the stimuli used here^18^. As can be seen (Fig. 5a), the receptor responses modeled by the standard TS model decreased in both spatial extent and absolute numbers as either phase or spatial dispersion increased. These trends can be depicted by summing the spikes produced by the model across each afferent family and averaging in time to produce mean firing rate (Fig. 5b). These simulation results correlate negatively with subject data collected here (Fig. 5c). Although the firing rates predicted by TS would not appear to account for our data, it is evident in Fig. 5a that both phase and dispersion have a systematic effect on the spatial distribution of responses. To test whether temporal or spatial variation in afferent response might encode intensity, we applied Gabor filters^4,25,26^ (details in ‘Methods’) but did not find a strong positive correlation with our roughness data at the optimal Gabor parameters found in the cited studies.

A striking feature of Fig. 5a, however, is the wide scatter of responses at low phase, and the near elimination of this scatter at 180 degrees phase. Of relevance, TS models the skin mechanics with both a quasistatic component based on the stresses produced by pin indentation of an elastic half space, and a dynamic component based on surface wave propagation^18^. We speculated that the dynamic component may have contributed to the phase dependence of scatter because the wavelength is long (200mm at 40Hz, 100mm at 80Hz) relative to the pin spacing. Thus, out-of-phase motion will cause destructive interference, reducing the amplitude of the dynamic motion, especially in the far field. To test this, we disabled the wave propagation mechanism in the TS software and repeated the simulations. We found that while SA-I and PC rates did not predict roughness judgments (SA-I: overall r = 0.19, PC: overall r = − 0.18) the RA rates did (Fig. 5d) with overall r = 0.88 (Fig. 5d). SA-I rates were a poor predictor because these receptors were excited only at the level near their baseline in static indentation. PC rates were a poor predictor because they were heavily influenced by stimulation frequency. Thus, only the firing rates of the RA afferents were able to correlate well with the observed dependence of perceived roughness on phase and dispersion, and only when the wave propagation mechanism was disabled. One explanation for this may be that TS over-estimates the effect of wave propagation on RA activation. While there is strong evidence that PC receptors respond to wave propagation in the skin, even over long distances^27–29^, there is considerably less evidence that RA receptors respond as strongly^30^. It may be the case that PCs respond to particle motion (e.g., acceleration), while RAs respond principally to skin strain which scales as particle motion divided by wavelength. As mentioned above, the wavelengths are quite long relative to the sizes and spacings of the pins; as such, it is possible that RAs respond weakly or not at all to wave propagation on the skin. This is a topic that bears closer examination in future studies.

## Conclusion

Our results provide evidence that perceived roughness of surfaces, which generate high frequency excitation of skin tissue, may be encoded by variability in micron-scale deformation and stretch created around the relatively coarsely-spaced mechanorecep- tors. In a similar vein, surface variations at various length scales may be reproduced by a more coarsely defined but nonetheless high-bandwidth array stimulation within the fingerpad. The observed sensitivity of threshold values to the spatial pattern implies that even smaller phase delays, and thus the rendering of finer spatial detail, may be achieved under optimal pin head size and spacing. Moreover, extrapolation of threshold phase values found in exp. 2 (Fig. 3) and the robust trend of roughness scaling in exp. 3 (Fig. 4), would suggest that sensitivity to the spatial profile of skin indentation is significant at frequencies well beyond 80Hz. These observations may explain the finding that skin vibration alone cannot account for the perceptual qualities of fine natural textures, necessitating within-fingerpad skin excitation^7^. Furthermore, a wide-band summary mechanism that is not frequency dependent may also explain the invariance of perception to touch conditions, such as fingertip speed and pressure, that tend to strongly affect vibrations of skin^6,9^.

Future research should continue to explore spatially distributed, wide-bandwidth fingerpad excitation to further the understanding of texture perception and guide the development of technology that can achieve its realistic display under the fingertip. One important topic is the spatial limit of perceptability in distributed information (typical estimates of this value are on the order of 1 mm^4^). A second is the perceptual threshold of displacement as stimuli are rendered at increasingly fine spacing. A third is the upper frequency at which the spatial distribution of fingerpad excitation is perceptually relevant. A fourth is the effect of pin distribution and variable phase between pins on higher-level percepts beyond roughness. Finally, compact new devices need to be developed that will operate at the limits of perception, and these should be applied to the engineering challenge of texture capture and playback.

## Methods

### Apparatus

**Figure 6:**
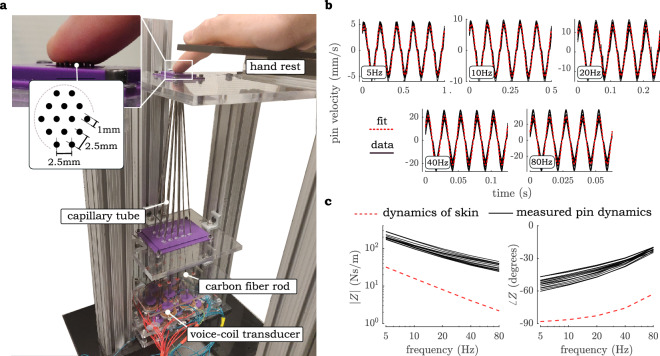
Experiment hardware and its characterization. (**a**) Pin array vibrotactile apparatus used in this study. (**b**) Vertical motion of each of the 14 pins measured by an LDV as they were actuated by sinusoidal currents across the frequency range used in the psychophysical study. (**c**) Measured impedance magnitude and phase of 14 pins relative to that of the finger’s assumed dynamics. Note that the measured pin phase does not appear to be predicted by a linear dynamic model where. ∠*Z*(*ω*)*|*_*ω→*0_ = *−* 90 due to the nonlinear contributions of static friction.

A pin array actuator (Fig. 6a) capable of broadband actuation was developed to study the influence of spatially discretized displacement of skin tissue within the fingerpad on perception. The array was composed of 14 individually driven pins made up of 400mm long and 1mm thick carbon fiber rods which are moved translationally by voice coil actuators (TEAX19C01-8). Each of the rods was fixed to a voice-coil via an additively manufactured component designed to appropriately angle the rod through a steel capillary tube channel, with an inner diameter of 2 mm, to the array bed. The inside of each capillary tube was filled with polymer grease to mitigate static friction forces contributing non-linearity in the force/displacement relationship of pin dynamics and to damp out resonant system response. Voice coils driving each pin were stacked closely side by side on two levels of acrylic plates to minimize off-axis motion of the pins. The maximum angle of rod motion relative to the normal axis of the array bed and thus the contacting fingertip skin was 7°.

Pin displacement was driven by signals generated from a series of 8bit DACs (MCP4902) and updated at 4 kHz using a PIC-32 microcontroller. Each of these signals was amplified by a transconductance current amplifier designed around a high power op-amp (OPA552) with an overall transconductance gain of − 0.1 A/V. Amplifiers could supply maximum currents of ± 0.3 A, which produced vertical peak-to-peak motion of the pins of up to 0.33 mm (Fig. 6b). The velocity of each pin as it was driven by sinusoidal current was measured using an LDV (Polytec IVS-500). We found good agreement between exact sinusoidal motion and the measured pin motion produced by the input sinusoidal force R^2^ = 0.98 ± 0.02. Slight deviations occurred at the extremes of pin position where static friction was engaged.

One of the chief design considerations of the apparatus was the controllability of pin motion under arbitrary loads provided by the contacting fingertip. In order to guarantee that pin motion remained consistent throughout the experiment, we selected and engineered the dynamics of the voice-coil array system to exhibit impedances considerably larger than that of the finger while guaranteeing sufficient range of motion under power constraints of the transducer. The measured impedance response compared to that of the bulk skin of the finger is shown in Fig. 6c. Fingertip impedance values used in the figure were adapted from skin parameters found in another study^31^. This impedance model is expected to be a significant overestimation (> 10 times) since it reflects impedances of the entire fingerpad whereas the apparatus engages only a partial area of skin with each individual pin loaded by a fraction of total normal force applied by the fingertip. We find that the impedance of each pin is about 5 times that of the bulk skin, and with consideration for local impedances we expect that additional contributing fingertip impedance will not significantly impact pin motion relative to an unloaded regime. Characterization of dynamics revealed some deviation in phase of pin motion relative to a second order system model. This deviation on the order of 30° can be attributed to the static friction forces affecting each pin inside the capillary tube. All pins appear to be affected by friction similarly, however, and the contribution of friction to pin motion should have no effect on the scope of the current study.

We constrained the frequency content of a given stimulus used in this study to a single frequency selected within the 5–80 Hz range. The upper frequency limit was selected based on dynamic considerations of skin tissue, since at > 80 Hz skin tends to decouple from the transducer head due to the dynamic phase lag occurring with increasingly dominant damping components^31^. In this device this phenomenon may have contributed to a loss of control in the skin displacement profile. We thus constrained skin actuation within the frequency range where dynamic response is dominated by stiffness and where minimal decoupling should occur.

### Participant experiments

Thirteen participants aged 20–28 took part in the psychophysical experiments conducted here, with 10, 10, and 11 participants assigned to the first, second, and third experiments respectively (distributed in a way as to limit each subject’s total time commitment to an hour). Experiments were approved by the Northwestern University IRB and were performed in accordance with relevant guidelines and regulations. Participants gave informed consent, and they were financially compensated for their time. All participants reported to be right-handed and all used their dominant hand during judgment tasks. Prior to taking part in the experiments, participants washed their hands with soap and warm water and dried them with a paper towel. They were instructed to wear active-noise cancelling headphones the generated pink noise to mask audio cues generated by the device. Participants had no view of the array during the experiment; thus, no visual cues of the stimulus were available.

All stimuli were rendered at one of five frequencies: {5, 10, 20, 40, 80} Hz. During training and experiments, the pin displacement was selected such that all stimuli were of similar perceptual intensity as judged by the lead author. Pin velocity amplitudes were set to {4, 5, 6, 7, 7} mm/s at each respective frequency, which corresponds to displacement amplitudes of {254, 160, 96, 56, 28} µm peak-to-peak.

During experiments, the participant’s palm of the dominant hand was kept flat on a hand rest positioned over the apparatus with their index finger extended out and placed onto the array. The non-dominant hand was used to interact with the GUI. The finger made contact with the array bed at ≈ 30° angle of incidence. Only the fingerpad received stimulation during the experiments, and the participant was otherwise completely decoupled from any vibrations produced by the device. The interface between the finger and pin array was monitored by the experimenter to ensure that the skin made static contact with all of the pins.

A training session preceded the experiments. Using a GUI, which was later also used in experiments, participants could choose to render one of three available patterns under their fingertip. One of these patterns was an odd one out and two were the same stimulus. When ready, participants were to select the odd stimulus. During this session, but not during experiments, correct stimulus choices were revealed after a selection was made. Ten trials were conducted under this protocol spanning the range of stimuli used in the experiments.

### Experiment 1

In the first experiment participants were tested under a 3AFC protocol of stimulus identification. Upon being presented with three stimuli, two of which were the same, they were asked to identify the odd one out. The aim of this experiment was to find the cutoff frequency past which participants could no longer identify specific stimulation points at the fingerpad but rather encoded tactile information in summary fashion across all points of excitation. To that end, they were tested on the discriminability of stimuli: S1 vs. S2A and S2A vs. S2B, as they are shown in Fig. 1. S1 and S2A stimuli differed in both the shape of pin motion as well as the summary time-based spatial distribution of thereof, whereas S2A and S2B differed only in shape (movement direction of the indentation across the skin). The first comparison served as a baseline for discriminability across the frequency range to find whether deviation from uniform skin displacement was perceptible. The second was aimed specifically at finding the cutoff frequency at which shape information of the stimulus could be no longer perceived. Participants made their choices 5 times per frequency, across two tests, totaling 50 trials.

### Experiment 2

In the second experiment we performed a parametric adaptive procedure under 3AFC protocol with the goal of obtaining the perceptual threshold associated with deviation from synchronization of pin motion. To that end, we compared S1 vs. S3-*x* and S1 vs. S5-*x* (Fig. 1), where *x* represents phase delay in degrees between two pre-selected groups of pins. Note that when *x* = 0 the four stimuli are equivalent and this point serves as the ’origin’ of spatio-temporal space with which we describe summary-based pin motion parameters. To accurately probe the characteristics of this space, the second experiment was performed at frequencies where spatially distributed pin motion was perceived in a summary fashion, i.e. 40 Hz and 80 Hz, and the shape of displacement profile itself could not cue the subjects. The first comparison, S1 vs S3-*x*, was performed with all pins actuated at 40 Hz but phased by *x*, and the second, S1 vs S5-*x*, was performed with all pins actuated at either 40 Hz or 80 Hz but phased by *x*. Since the S3 and S5 stimuli are different in the spatial dispersion of pin grouping, comparison of thresholds in discrimination across these two stimuli can be used to deduce the effect of spatial distribution on sensitivity to deviation from uniform motion. Stimulus presentation in this experiment was interleaved across the three separate adaptive procedures.

During the experiment, phase delays (*x* values) were adaptively selected based on the method of maximum likelihood estimation (MLE) aimed at sampling the mid-range, P(*x*)|_*x→x*_*∗* = 0.66, of the assumed psychometric curve following a logistic model with lower and upper bounds of P(*x*)|_*x→*0_ = 0.33 and P(*x*)|_*x→*∞_ = 1.0. Our simulations revealed that an individual participant’s deviation from the prior psychometric slope did not significantly impact estimation of the threshold values, in line with previous research on applying MLE toward psychophysics^32,33^. The slope of the psychometric function assumed in the experiment was adapted from a longer study performed with the lead author using a method of constant stimuli in 200 trials. Although the final, estimated threshold values are reported as modes of the posterior likelihood distribution, the stimulus selection strategy during experimentation itself involved sampling the mean of concurrent likelihood distributions of threshold probability, starting with *x* = 90° as the first phase delay to be tested. Through simulation we found this strategy to yield faster convergence to the true threshold compared to sampling based on estimated mode as is often performed in MLE algorithms. This is likely because the extremes of the psychometric curve, which provide the least information regarding the participant’s perceptual threshold, were altogether avoided.

### Experiment 3

Prior to this experiment, participants were presented several textiles upon which roughness judgments were made as a way of reinstating the concept. No feedback was given regarding their objective performance in the training. Immediately following this training period, participants performed a free magnitude estimation of the associated roughness quality of 12 stimuli with different spatial distributions and timing delays, rendered at 40 Hz and 80 Hz, totaling 24 estimations in total. Stimuli were composed of S3-*x*, S4-*x*, and S5-*x* spatial patterns with *x* = {45, 90, 135, 180} degrees. Stimulus presentations were randomized in order and participants were not restricted in time when making the judgment.

### TouchSim

We used the TS software to simulate responses of the three afferent types (SA-I, RA, and PC) that innervate the distal phalanx in response to the stimuli used in Experiment 3. All simulations were run for 10 sec. and repeated 20 times with different initial distributions of afferents. We found the distribution had a significant effect on summary afferent response characteristics, prompting multiple runs. The first set of simulations was performed at 1.2 mm base skin indentation (which corresponds to 1N normal load distributed on the skin assuming elastic modulus of skin to be 50kPa) using the same stimulation amplitudes used in the experiment, namely 56 µm and 28 µm at 40 Hz and 80 Hz respectively, and applying the standard TS model. The second set of simulations was performed with the wave propagation portion of TS disabled, leaving only static mechanics of skin deformation. This was done by decreasing wave propagation velocity within TS software by multiple orders of magnitude. We analyzed the simulated afferent spike trains using measures of intensity/roughness found in the literature: rate, temporal variation, and spatial variation. All the steps closely mimicked those applied in^4,26^, and we found no strong predictive power of temporal and spatial variance when using optimal parameters that were found in the cited studies. Maximum predictive power of variation was achieved when Gabor filters were generated using parameters closely matching the stimulus (2.5mm pin spacing, 12.5/25msec periods); nonetheless, these predictors still performed worse than RA rate in the absence of wave propagation.

## Data Availability

The datasets used and/or analysed during the current study available from the corresponding author upon request.

## References

[1] Katz D, Krueger LE (2013). The World of Touch.

[2] Hollins M, Risner SR (2000). Evidence for the duplex theory of tactile texture perception. Percept. Psychophys..

[3] Connor CE, Hsiao SS, Phillips JR, Johnson KO (1990). Tactile roughness: Neural codes that account for psychophysical magnitude estimates. J. Neurosci..

[4] Connor CE, Johnson KO (1992). Neural coding of tactile texture: Comparison of spatial and temporal mechanisms for roughness perception. J. Neurosci..

[5] Weber AI (2013). Spatial and temporal codes mediate the tactile perception of natural textures. Proc. Natl. Acad. Sci..

[6] Manfredi LR (2014). Natural scenes in tactile texture. J. Neurophysiol..

[7] Grigorii RV, Klatzky R, Colgate E (2021). Data-driven playback of natural tactile texture via broadband friction modulation. IEEE Trans. Hapt..

[8] Wiertlewski M, Lozada J, Hayward V (2011). The spatial spectrum of tangential skin displacement can encode tactual texture. IEEE Trans. Rob..

[9] Boundy-Singer ZM, Saal HP, Bensmaia SJ (2017). Speed invariance of tactile texture perception. J. Neurophysiol..

[10] Bensmaïa S, Hollins M (2005). Pacinian representations of fine surface texture. Percept. Psychophys..

[11] Wiertlewski, M., Hudin, C. & Hayward, V. On the 1/f noise and non-integer harmonic decay of the interaction of a finger sliding on flat and sinusoidal surfaces. In *2011 IEEE World Haptics Conference*, 25–30 (IEEE, 2011).

[12] Janko M, Primerano R, Visell Y (2015). On frictional forces between the finger and a textured surface during active touch. IEEE Trans. Hapt..

[13] Vega-Bermudez F, Johnson K (1999). Surround suppression in the responses of primate sa1 and ra mechanoreceptive afferents mapped with a probe array. J. Neurophysiol..

[14] Killebrew JH (2007). A dense array stimulator to generate arbitrary spatio-temporal tactile stimuli. J. Neurosci. Methods.

[15] Holliins M, Faldowski R, Rao S, Young F (1993). Perceptual dimensions of tactile surface texture: A multidimensional scaling analysis. Percept. Psychophys..

[16] Mountcastle VB, LaMotte RH, Carli G (1972). Detection thresholds for stimuli in humans and monkeys: Comparison with threshold events in mechanoreceptive afferent nerve fibers innervating the monkey hand. J. Neurophysiol..

[17] Saal HP, Wang X, Bensmaia SJ (2016). Importance of spike timing in touch: An analogy with hearing?. Curr. Opin. Neurobiol..

[18] Saal HP, Delhaye BP, Rayhaun BC, Bensmaia SJ (2017). Simulating tactile signals from the whole hand with millisecond precision. Proc. Natl. Acad. Sci..

[19] Johansson RS, Vallbo ÅB (1983). Tactile sensory coding in the glabrous skin of the human hand. Trends Neurosci..

[20] Sripati AP, Bensmaia SJ, Johnson KO (2006). A continuum mechanical model of mechanoreceptive afferent responses to indented spatial patterns. J. Neurophysiol..

[21] Corniani G, Saal HP (2020). Tactile innervation densities across the whole body. J. Neurophysiol..

[22] Fleming MS, Luo W (2013). The anatomy, function, and development of mammalian a *β* low-threshold mechanoreceptors. Front. Boil..

[23] Takahashi-Iwanaga H, Shimoda H (2003). The three-dimensional microanatomy of Meissner corpuscles in monkey palmar skin. J. Neurocytol..

[24] Kim SS, Sripati AP, Bensmaia SJ (2010). Predicting the timing of spikes evoked by tactile stimulation of the hand. J. Neurophysiol..

[25] Goodman JM, Bensmaia SJ (2017). A variation code accounts for the perceived roughness of coarsely textured surfaces. Sci. Rep..

[26] Lieber JD, Xia X, Weber AI, Bensmaia SJ (2017). The neural code for tactile roughness in the somatosensory nerves. J. Neurophysiol..

[27] Andrews J, Adams M, Montenegro-Johnson T (2020). A universal scaling law of mammalian touch. Sci. Adv..

[28] Libouton X, Barbier O, Berger Y, Plaghki L, Thonnard J-L (2012). Tactile roughness discrimination of the finger pad relies primarily on vibration sensitive afferents not necessarily located in the hand. Behav. Brain Res..

[29] Manfredi LR (2012). The effect of surface wave propagation on neural responses to vibration in primate glabrous skin. PLoS ONE.

[30] Gardner EP, Palmer CI (1989). Simulation of motion on the skin. i. receptive fields and temporal frequency coding by cutaneous mechanoreceptors of optacon pulses delivered to the hand. J. Neurophysiol..

[31] Wiertlewski M, Hayward V (2012). Mechanical behavior of the fingertip in the range of frequencies and displacements relevant to touch. J. Biomech..

[32] Shen Y, Richards VM (2012). A maximum-likelihood procedure for estimating psychometric functions: Thresholds, slopes, and lapses of attention. J. Acoust. Soc. Am..

[33] Green DM (1990). Stimulus selection in adaptive psychophysical procedures. J. Acoust. Soc. Am..

